# Machine learning for predicting hepatitis B or C virus infection in diabetic patients

**DOI:** 10.1038/s41598-023-49046-9

**Published:** 2023-12-06

**Authors:** Sun–Hwa Kim, So–Hyeon Park, Heeyoung Lee

**Affiliations:** 1 Department of Clinical Medicinal Sciences, Konyang University, Nonsan, Republic of Korea; 2 College of Pharmacy, Inje University, Gimhae, Republic of Korea

**Keywords:** Medical research, Risk factors

## Abstract

Highly prevalent hepatitis B and hepatitis C virus (HBV and HCV) infections have been reported among individuals with diabetes. Given the frequently asymptomatic nature of hepatitis and the challenges associated with screening in some vulnerable populations such as diabetes patients, we conducted an investigation into the performance of various machine learning models for the identification of hepatitis in diabetic patients while also evaluating the significance of features. Analyzing NHANES data from 2013 to 2018, machine learning models were evaluated; random forest (RF), support vector machine (SVM), eXtreme Gradient Boosting (XGBoost), and least absolute shrinkage and selection operator (LASSO) along with stacked ensemble model. We performed hyperparameter tuning to improve the performance of the model, and selected important predictors using the best performance model. LASSO showed the highest predictive performance (AUC-ROC = 0.810) rather than other models. Illicit drug use, poverty, and race were highly ranked as predictive factors for developing hepatitis in diabetes patients. Our study demonstrated that a machine-learning-based model performed optimally in the detection of hepatitis among diabetes patients, achieving high performance. Furthermore, models and predictors evaluated from the current study, we expect, could be supportive information for developing screening or treatment methods for hepatitis care in diabetes patients.

## Introduction

Diabetes mellitus (DM) has remained one of the most problematic chronic metabolic disorders in humans over the past decades^1^. Based on the recent statistics released by the centers for disease control and prevention (CDC), DM affects approximately 34.2 million individuals in the US^2^. DM can be primarily categorized into two types such as type 1 (T1DM) and type 2 diabetes mellitus (T2DM)^3^. T1DM is an immune-mediated disease leading to absolute deficiency of endogenous insulin caused by β-cell loss in the pancreas^3^. On the other hand, T2DM is a prevalent endocrine disorder characterized by multifactorial mechanisms^3^. These mechanisms encompass insulin resistance, increased glucose production by the liver, and impaired insulin secretion^3^. In both T1DM and T2DM, a combination of genetic and environmental factors can lead to the gradual decline of β-cell mass and/or function, which is clinically manifested as hyperglycemia in T1DM and T2DM^4^. Once hyperglycemia occurs, patients with any forms of diabetes are susceptible to developing complications caused by impact on several organ systems over time^4^. Relevant to disturbed glucose homeostasis, hepatitis B virus (HBV) and hepatitis C virus (HCV) infections in populations with DM have recently been reported as an emergent comorbidity^5,6^. With a considerably high prevalence, 865 outbreaks of HBV infection were previously reported among adults who were diagnosed with diabetes^5^. Adults with DM have a 60% higher prevalence of HBV infection and are twice as likely to experience acute HBV infection compared to adults without DM^7^. According to the study by Gisi et al.^8^, along with HBV, the prevalence of HCV in the diabetic group was significantly higher than that in the non-diabetic group.

While there is a high prevalence of HBV or HCV infections in diabetes, regarding asymptomatic nature in hepatitis, the challenges associated with screening in some vulnerable populations such as diabetes patients are still remained. Some patients have reported never developing histologic evidence of liver disease even after decades of infection reflecting obstacles in identifying hepatitis^9,10^. Furthermore, in those with comorbid conditions such as DM, identifying or predicting HBV or HCV accurately has proven to be also challenging, emphasizing the need for more selective screening methods^11–14^. Moreover, previous studies have yielded conflicting results regarding the important risk factors for hepatitis development in individuals with diabetes^15,16^. Therefore, further studies conducted with improved strategies such as using machine learning models are necessary to provide essential information about predictors of hepatitis development in diabetes, aiding clinical decision-making.

In response to these unmet needs, machine learning has emerged as a promising alternative to traditional hepatitis screening strategies in recent years, making significant breakthroughs in the realm of public screening even including finance or wireless sensor network (WSN)^17–21^. In finance and WSNs that without clinical data, after tuning hyperparameters, machine learning revealed the best results among other base learning models^18,21^. Besides, given the demonstrated effectiveness of machine learning models across various fields, especially in healthcare, machine learning has emerged as a powerful approach. It enables the extraction of valuable information from imbalanced clinical datasets and facilitates decision-making through accurate predictions^17^. Doğru et al.^19^ using clinical data showed the best accuracy in predicting early diabetes risk through hybrid super ensemble learning model (99.6%). Furthermore, several studies^22,23^ have been conducted on predictive machine-learning models for diabetes treatment using laboratory and clinical data. Ozyilmaz et al.^24^ demonstrated the possibility of accurate hepatitis identification through machine learning, and numerous previous works^25–29^ have consistently used machine learning to facilitate the early prediction of a significant number of people who are at high risk for hepatitis. Yağanoğlu et al.^26^ even tried to develop a model with high performance in predicting HCV with HCV dataset by comparing eight machine learning models, such as random forest (RF) and K-nearest neighbor, which yielded an overall accuracy of 96.75%. Another previous study based on clinical records of 155 hepatitis B patients predicting HBV revealed adaptive boosting as an accurate model (92%) for diagnosis after comparing other machine learning models such as eXtreme Gradient Boosting (XGBoost) and RF^29^. Given the significant previous results achieved by individual machine learning models in predicting hepatitis, to enhance predictive performance, ensemble learning has been applied for various researches as well^18–20,30,31^. By integrating various machine learning algorithms, ensemble techniques aim to make more accurate predictions compared to a single classifier^30^. When the base models are diverse and independent resulted in lower accuracy of predictions, regarding as primary goal, using ensemble models is to reduce generalization errors^18^. Despites of generally improving performance of ensemble models, several researches^25,31^ in different study settings still showed lower accuracy value of ensemble learning rather than a single base model. Edeh et al.^25^ still showed that an artificial intelligence-based ensemble model could predict HCV with an accuracy of approximately 94% for early diagnosis and treatment for HCV infection. Based on these discrepancies, we need to explore optimal performance metrics in single and ensemble models for predicting hepatitis development in DM patients.

To the best of our knowledge, there has not been a study evaluating machine-learning models, including ensemble models, for predicting hepatitis development exclusively among patients with diabetes. In this study, we have conducted an investigation to determine the most suitable machine-learning models and identify relevant risk factors.

## Results

### Characteristic analysis

**Figure 1:**
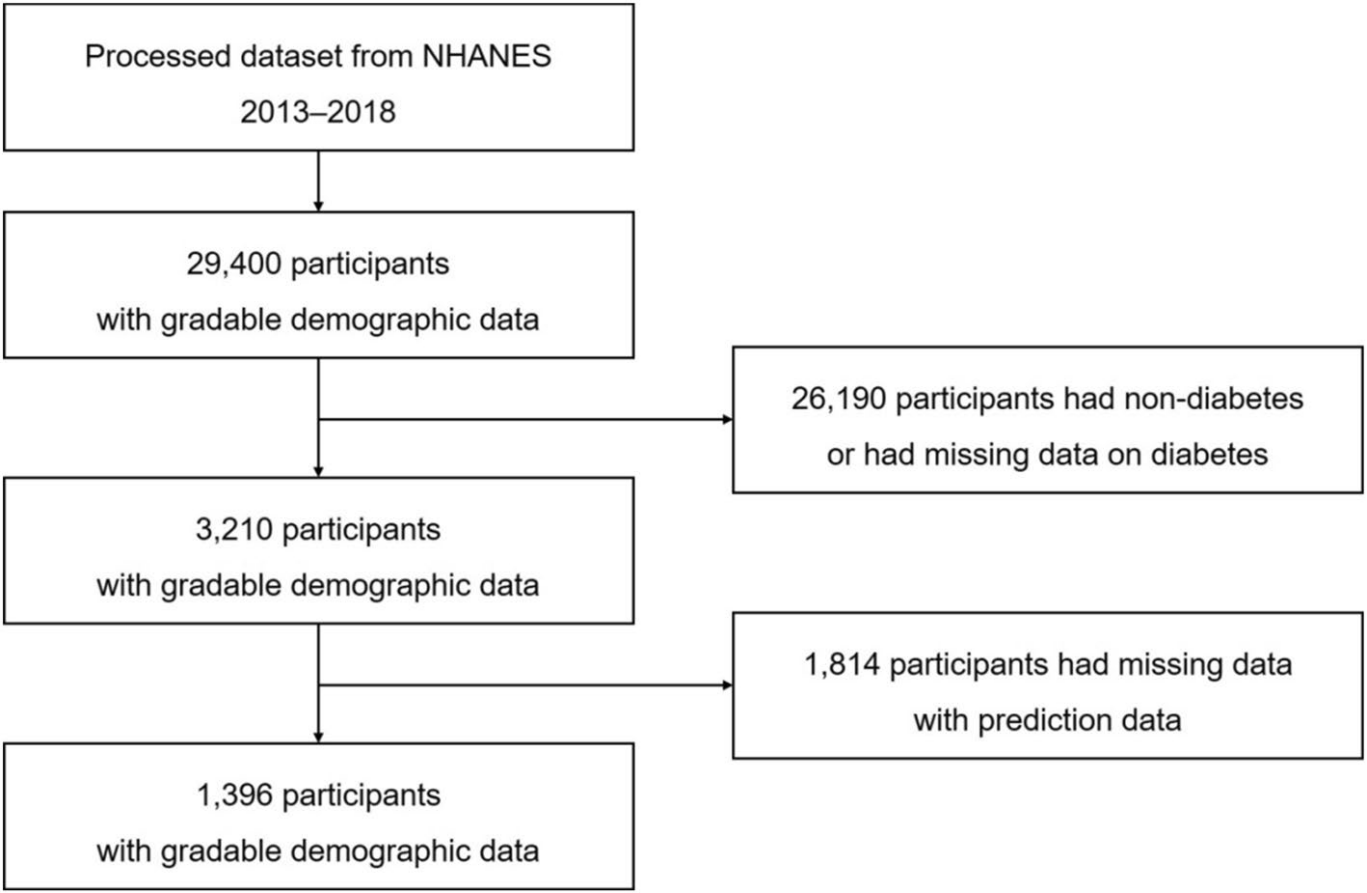
Flowchart of dataset creation.

We evaluated the demographics, body measurements, lipids, and questionnaire data to analyze the association between diabetes and the 12 risk factors for hepatitis. The dataset that was preprocessed from the National Health and Nutrition Examination Survey (NHANES) 2013–2018 included 29,400 participants. A total of 26,190 participants without diabetes or missing data on diabetes were excluded. Consequently, a total of 3210 diabetes identification cases remained in the complete dataset. Thus, a total of 1396 diabetic patients were included in the study (Fig. 1). The synthetic minority oversampling technique (SMOTE) balancing technique was applied to the dataset prior to establishing the model owing to the imbalanced ratio of non-hepatitis to hepatitis patients. Following data normalization, the machine-learning methods were applied to train and test the models on the training and test datasets.

A total of 1396 people with a mean age of 54.66 participated in the study, including 64 with HBV or HCV and 1332 without HBV or HCV (Table 1). The hepatitis group had a higher percentage of non-Hispanic White and Asian individuals, whereas the non-hepatitis group had a higher percentage of Mexican and other Hispanic individuals. Males comprised 70.3% of the patients in the hepatitis group. More than half of the patients in the hepatitis group administered illegal drugs.

**Table 1:** Characteristics of the study population.

Variables	Total (n = 1396)	Hepatitis B or C (n = 64)	No hepatitis B or C (n = 1332)
Demographics			
Age, mean ± SD	54.66 ± 10.85	57.69 ± 8.423	54.52 ± 10.93
Male, n (%)	717 (51.36)	45 (70.3)	672 (50.5)
Race, n (%)			
Mexican American	269 (19.27)	7 (10.94)	262 (19.67)
Other Hispanic	159 (11.39)	4 (6.25)	155 (11.64)
Non-Hispanic White	400 (28.65)	23 (35.94)	377 (28.30)
Non-Hispanic Black	375 (26.86)	17 (26.56)	358 (26.88)
Non-Hispanic Asian	135 (9.670)	10 (15.63)	125 (9.384)
Poverty, mean ± SD	2.393 ± 1.603	2.006 ± 1.651	2.411 ± 1.599
Body measure			
BMI, mean ± SD	111.2 ± 7.934	31.45 ± 7.128	33.55 ± 7.961
Waist circumference, mean ± SD	122.0 ± 17.32	107.8 ± 16.19	111.3 ± 17.36
Lipids (mg/dL)			
HDL cholesterol, mean ± SD	47.78 ± 14.93	46.27 ± 13.39	47.85 ± 15.00
Total cholesterol, mean ± SD	188.1 ± 49.21	168.4 ± 41.73	189 ± 49.36
Questionnaire data			
Receiving blood, n (%)	184 (13.18)	15 (23.44)	169 (12.69)
Hepatitis B vaccine, n (%)	430 (30.80)	14 (21.88)	416 (31.23)
General health condition, n (%)	781 (55.95)	32 (50)	749 (56.23)
Illegal drug injection, n (%)	36 (2.579)	23 (35.94)	13 (0.976)
Diagnosis			
Hepatitis B, n (%)	26 (1.862)	26 (40.63)	0
Hepatitis C, n (%)	40 (2.865)	40 (62.5)	0

BMI, body measure index; HDL-cholesterol, high-density lipoprotein cholesterol.

### Model performance comparison

We used a randomized search with ten iterations threefold cross-validation for each of the four models to predict hepatitis. Single machine learning models including RF, XGBoost, support vector machine (SVM), and least absolute shrinkage and selection operator (LASSO) algorithm and stacked ensemble model were created. Sixteen statistically significant clinical parameters were included within these models. The performance comparison results of the four machine-learning methods, both before and after hyperparameter tuning, are presented in Table 2. The LASSO achieved the best accuracy value (0.978) after hyperparameter tuning. The sensitivity of all four models was generally low. Specificity of LASSO was also higher than other models (0.993). Additionally, the precision and F1 score of LASSO surpassed those of the other three models. Consequently, LASSO outperformed the other models across all evaluation metrics as presented in Table 2. To improve the results, we applied the stacking ensembles algorithm to combine the predictions of individual models. In the stack-based ensemble model, the accuracy is 0.945, without showing a significant improvement compared to individual models. The specificity was 0.958, and the sensitivity was 0.500, indicating that it did not outperform the best individual model, in terms of predictive performance.

**Table 2:** Performance of algorithms in diagnosis based on with and without hyperparameter tuning.

Algorithm	Sensitivity	Specificity	Precision	F1 score	Accuracy
Without hyperparameter tuning					
RF	0.683	0.903	0.857	0.802	0.760
SVM	0.791	0.916	0.889	0.837	0.859
XGBoost	0.705	0.882	0.836	0.800	0.765
LASSO	0.591	0.898	0.831	0.756	0.691
With hyperparameter tuning					
RF	0.461	0.978	0.400	0.429	0.962
SVM	0.500	0.990	0.600	0.545	0.976
XGBoost	0.500	0.968	0.316	0.387	0.954
LASSO	0.500	0.993	0.667	0.571	0.978

RF, random forest; SVM, support vector machine; XGBoost, extreme gradient boosting; LASSO, least absolute shrinkage and selection operator.

**Figure 2:**
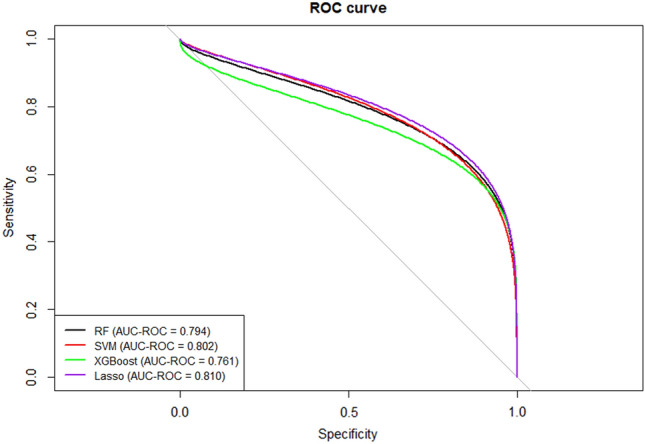
Performance of algorithms in diagnosis.

The ROC curve for the classification performance of the four machine-learning models is depicted in Fig. 2. The LASSO model performed the best among all of the classifiers, with an area under the receiver operating characteristic curve (AUC-ROC) of 0.810. The AUC-ROC scores of the RF (0.794) and XGBoost (0.761) were lower than that of the LASSO.

### Feature importance

**Figure 3:**
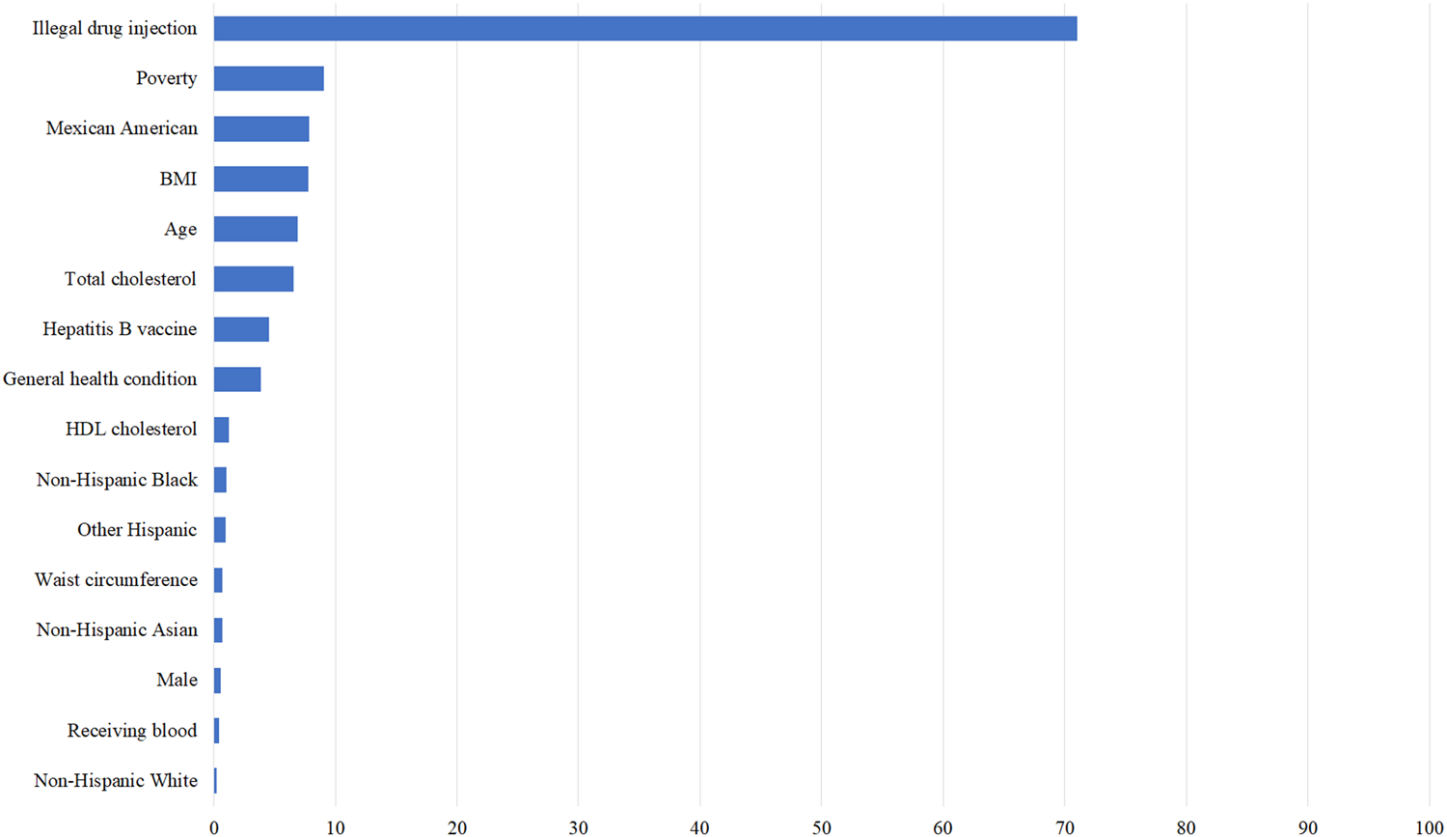
Ranked importance scores.

The importance results for the 16 variables based on the highest-performing LASSO model are presented in Table 3 and Fig. 3. The most important predictor was illegal drug injection (71.036), which was the most reliable variable, and this was closely followed by the ratio of family income to poverty (9.084), Mexican Americans (7.867), the body mass index (BMI) (7.798), and age (6.930). Finally, non-Hispanic White (0.218) were placed in the lowest ranking.

**Table 3:** Ranked importance scores.

No	LASSO	
	Variable	Importance score
1	Illegal drug injection	71.036
2	Poverty	9.084
3	Mexican American	7.867
4	BMI	7.798
5	Age	6.930
6	Total cholesterol	6.538
7	Hepatitis B vaccine	4.565
8	General health condition	3.877
9	HDL cholesterol	1.235
10	Non-Hispanic Black	1.057
11	Other Hispanic	1.001
12	Waist circumference	0.731
13	Non-Hispanic Asian	0.681
14	Male	0.561
15	Receiving blood	0.401
16	Non-Hispanic White	0.218

BMI, body measure index; HDL-cholesterol, high-density lipoprotein cholesterol.

## Discussion

This study evaluated machine-learning models used in previous clinical studies to demonstrate the best predicting model and predictors for HBV or HCV infection in patients with DM. The results revealed that all models exhibited improved performance after the hyperparameter tuning process, with LASSO demonstrating the highest predictive ability for HBV or HCV infection development in diabetic patients. Hyperparameter optimization involves selecting the most suitable parameter values from a given parameter space, allowing for the optimization of model complexity and enhancing the performance of machine learning algorithms^32^. Hyperparameter optimization allows for the improvement of algorithm performance by identifying the correct parameter values, as they cannot be directly learned from the algorithms themselves^32^. Through hyperparameter optimization, LASSO emerged as the best-performing model in our study. Previously, LASSO has already demonstrated its clinical efficacy through several studies^21,33–36^. Ahn et al.^34^ indicated that LASSO showed superior performance in predicting hepatocellular carcinoma in patients with chronic hepatitis B, particularly when utilizing twelve-month post-treatment parameters (AUC-ROC = 0.843). In another study focusing on the prediction of hepatocellular carcinoma, LASSO demonstrated excellent predictive performance when combined with first-year clinical parameters in chronic hepatitis B patients^35^. This highlights the accurate risk assessment and emphasizes the potential of LASSO for early detection in hepatocellular carcinoma prediction. Other previous studies^36,37^ predicting diabetes also demonstrated that LASSO (0.84) has been shown to exhibit promising performance compared to other models such as RF, gradient boosted decision tree, deep neural network and the reference model using a logistic regression, providing evidence of its effectiveness in accurately predicting diabetes. Consequently, the current study demonstrates that the LASSO is even suitable for evaluating predictors of hepatitis in diabetic patients and highlights the importance of hyperparameter tuning in improving model accuracy. These findings provide valuable insights for developing more effective predictive models and aiding clinical decision-making.

In an attempt to demonstrate more improved performance, ensemble approach was tried in the current study, but did not achieve favorable results. One study demonstrated the efficacy of ensemble model by achieving the highest accuracy of 99.6% and superior ROC curve performances compared to other machine learning algorithms^19^. Buyrukoğlu et al.^20^ showed that the individual models exhibited lower performance, with an accuracy of approximately 95% compared to the ensemble model. Particularly, among the ensemble models, the artificial neural network indicated the highest accuracy of 99.1%. While several studies^19,20^ have shown better performance results using ensembles, other studies^31,32^ have presented lower performance metrics than single base models, similar outcomes to our research. A prior research using similar data to our study, NHANES, has shown that there were variations of accuracy in the performing results of ensemble models depending on diseases such as CVD (0.839) and DM (0.737) and period collect data^31^. For predicting DM, the performance metrics were lower than a single model^31^. In another study^32^, stacking ensembles combining models with individually excellent performance also showed lower performance results than base models such as XGboost, which is also consistent with our findings. Despites of combining high-performing models in our study, the ensemble results indicated lower performance compared to individual models. As consistently shown in previous studies, our study also demonstrated stacking ensemble always did not improve performance metrics for the predictions, suggesting the need for further future researches on ensemble performance in various settings or datasets.

The current study evaluated illegal drug injection, poverty, and race as key factors for predicting hepatitis. The results of major predictor variables related to hepatitis prevalence in diabetic patients, as observed in the study by Han et al.^38^, were consistent with the findings of our study. Illegal drug use occurs globally, negatively affects the quality of life of individuals and communities, reduces productivity, and significantly increases the demands on healthcare systems^39^. Parenteral exposure with the sharing of unsterilized needles is a risk factor for the transmission of viruses in illegal drug users. Additionally, it should be noted that over 25% of individuals with diabetes administer insulin^40^, which can potentially be transmitted if they share equipment, supplies, or insulin with others^41^. Furthermore, diabetes patients who are at a higher risk of experiencing elevated stress levels and immune dysfunction may also exhibit increased vulnerability to illicit drug use and transmission of HCV^38^. Moreover, multiple studies have reported a correlation between drug users and the development of HBV or HCV^2,42–47^. Eckhardt et al.^48^ indicated that 343 out of 714 participants in their study on young Americans who were injected with drugs were infected with HCV. Furthermore, the hepatitis population-attributable fraction in 2013 for HBV (10% in North America and 1% in Latin America) and HCV (81% in North America and 31% in Latin America) was further increased from 1990 for HBV (6% in North America and 1% in Latin America) and HCV (60% in North America and 19% in Latin America)^47^. Although insufficient evidence is available and controversies surround the effects of diabetes on HBV or HCV infections, and further research is required, previous studies have consistently demonstrated the impact of diabetes on HBV or HCV infections^49,50^. According to Schillie et al.^49^, a higher prevalence of HBV infection was observed among persons with diabetes compared to those without diagnosed diabetes (odds ratio (OR) = 1.60; 95% CI 1.30–1.90, *p* < 0.05). Furthermore, a meta-analysis study^50^. Revealed that patients with type 2 diabetes were at a higher risk of acquiring HCV infection than non-type 2 diabetic patients (OR = 3.50, 95% CI 2.54–4.82; I2 = 82.3%). Moreover, considering the problems or dysfunction of the immune system in diabetic patients, greater vulnerability to virus infection may be expected with the use of illegal drugs^51^. Therefore, this study can provide important insights in identifying a wide range of illegal drug users at high risk of HCV and HBV infection. By identifying and assessing predictive factors resulted from the current study, healthcare providers can intervene at an early stage to prevent or manage hepatitis infections in this specific population.

The next most relevant predictor in this study was poverty. People who live in poverty are generally vulnerable to infectious diseases owing to their poor living conditions and difficulties in accessing healthcare^52^. Although various factors contribute to poverty, such as age or education levels, and these are closely related to the spread of infectious diseases, poverty was only defined with the income ratio in this study^53^. In particular, based on the American Association for the Study of Liver Disease (AASLD), HBV and HCV are the leading infectious diseases that are closely related to poverty^54,55^. Greene et al.^56^ analyzed surveillance data in New York City and reported that chronic hepatitis C was included in diseases that were related to severe poverty in people with a low income and hepatitis B susceptibility rates are approximately 32% among individuals with a low income in Brazil^57^.

Moreover, previous studies have shown that diabetes is also associated with poverty^58^. The lack of primary healthcare owing to poverty makes it difficult for those living in poverty to access services that can reduce the incidence of hepatitis, especially in low-income countries^52^. Therefore, poverty in people with complications such as DM is a cause of increased HBV or HCV infections and is also likely to be an important factor in the incidence rate because of the cost burden^59^.

Race/ethnicity has historically been an important factor in many diseases. Although the mechanism did not clearly explain the outcomes of the current study, race is an important factor in the development of hepatitis in patients with DM. We demonstrated that Mexican Americans are highly affected races. Mexican Americans were the most important variable among the races. In previous studies, the HBV infection rate was estimated to be 2.9% for Mexican Americans, which reflects almost tenfold higher prevalence rate than that in the general population^60,61^. An increased percentage of HCV infection was observed among Mexican Americans during the more recent period (2011–2016) than from 1999 to 2010 (5.6% vs. 10.6%)^62^. This analytical evidence is consistent with the finding that Mexican Americans are significantly associated with infection. Furthermore, although our study did not demonstrated as highly important factors, HBV prevalence according to the NHANES, 20.5% of Asian Americans had been infected with HBV between 2011 and 2012^61^, which was also shown the association between the risk in Asian Americans and a high HBV or HCV prevalence rate in foreign-born Asian countries^63^. Regardless of the outcomes of the current study, more future studies with a larger scale data analyzing the prevalence of HCV and HBV in Asian Americans should be conducted based on this study.

Through identifying the contributing factors to disease manifestation, our findings indicate that machine learning models exhibit promising outcomes in the early detection of hepatitis among DM patients. Then, the current outcomes might contribute to model implementation in various tools such as web-based screening system, utilizing questionnaires to assess individuals' disease risk at developing hepatitis B or C. Thus, in clinical practice, with integrating survey and laboratory, models and predictors evaluated from the current study, we expect, could be supportive information for developing screening methods or treatment strategy for hepatitis care in DM patients.

Based on the encouraging findings of the current study, our research also exhibits several notable strengths. Firstly, to our knowledge, this study represents the first attempt to assess different machine learning models in predicting HBV and HCV infections in patients with DM. While a previous study examined the NHANES dataset and highlighted the susceptibility of DM patients to developing hepatitis^38^, our study contributes important insights for developing screening strategies and improving treatment accessibility in identifying vulnerable DM patients at risk of various infections. Secondly, we utilized various statistical analysis methods such as SMOTE method or hyper-parameter tuning to address the imbalanced data and evaluate the optimal machine learning model. Lastly, our study presented valuable insights into important predictors using machine learning models that exhibited remarkably high accuracy. Specifically, the LASSO model demonstrated the highest accuracy level, reaching nearly 98%. Using this highly performing machine learning model, our study successfully identified significant predictors for the development of hepatitis in patients with DM. By leveraging the superior performance of these machine learning models and the discovery of important factors, we can provide valuable information that can support the development of preventive care and policy targeting more vulnerable populations with DM.

Our study exhibits several limitations. First, the study was conducted on a population of Americans. Thus, different results may be obtained for populations from different countries or cultures. Although a machine-learning classifier was developed for use as an international instrument, it should be applied considering the culture, society, and environment of each country^64^. Therefore, future studies should include global data from other populations.

Second, as our dataset exhibited the cross-sectional nature of the NHANES dataset, it was difficult to determine the future prognosis of the patients. Although we predicted diabetes and related hepatitis at the time of investigation, longitudinal data are required to determine prognoses^65^.

Third, the lack of information among the participants may have led to the exclusion of several relevant results. Owing to the limited types of risk factors that were reported in other years and the small sample size, only 1396 people from the NHANES from 2013 to 2018 were selected for this study.

Fourth, although poverty is related to scholarity, data on education levels were not included in the current study. Considering the close correlation between scholarity and the spread of infectious diseases, it is important for the education level to be evaluated. However, the definition of education levels remains controversial and may affect the results^66,67^. Thus, we anticipate further studies on the impact of education levels on HBV or HCV infections among diabetic patients in the future.

Fifth, we evaluated HBV and HCV infections without serological data owing to the inconsistency of variables among the datasets and data unavailability. The NHANES collects data of participants including serological data, which are released in two-year cycles^68^. However, discrepancies in the variables were noted among the serological data collected from 2013 to 2018 for HBV. An additional variable in the laboratory results of the hepatitis B surface antigen, namely “indeterminate”, was provided for 2015 to 2018, which differed from the dataset that was collected from 2013–2014 that included only “positive” or “negative”. Furthermore, limited serological data for HCV infection could be accessed owing to the data restriction with low precision^68^.

Sixth, in the current study, we exclusively assessed existing machine learning models rather than undertaking the development of a new model. Through comprehensive searches of previous studies evaluating machine learning models predicting outcomes related to hepatitis, we finally selected four machine learning models shown higher performance metrics rather than other algorithms, which followed approach taken in a previous study^69^. Furthermore, it is important to note that surpassing the performance of established models falls beyond the scope of our research. In the future, we anticipate the development of novel machine learning models with the potential to enhance the accuracy of hepatitis prediction among diabetes patients. Developing new novel models improved interpretability and usability in clinical practice might contribute to the speed and accuracy of physicians' work, develop early diagnosis and treatment strategies, and improve screening protocols^70,71^. Finally, although the high prevalence of DM in patients with viral hepatitis is mediated by the development of liver cirrhosis^72,73^, liver cirrhosis data were not included in the current study. Liver ultrasound data, which provide objective measures for liver cirrhosis manifestations, were not released in 2013–2016. Therefore, further studies that include liver cirrhosis data should be conducted.

## Methods

### Dataset

The NHANES is a program of studies that is designed to assess the health and nutritional status of adults and children in the US. The NHANES questionnaire consists of demographic, socioeconomic, dietary, and health-related questions. A nationally representative sample of approximately 5000 individuals is gathered per year using this survey through a complex and multistage sampling design and the database is up-dated every two years. Furthermore, the NHAENS gathers data on 60 years and older, African Americans, and Hispanics to produce reliable statistics by reducing bias. Common public medical databases, such as the NHANES, provide researchers with important clues to the causes of diseases based on the distribution of health problems and risk factors in the population. All data are available for download from the NHANES website www.cdc.gov/nchs/nhanes/.

In this study, the NHANES data from 2013 to 2018 were used for the model validation and prediction of HBV or HCV in diabetic patients. We extracted the demographics, body measurements, lipids, and questionnaire data to analyze the association between diabetes and the 12 risk factors for hepatitis. The demographic data included age, gender (male), race (Mexican American, other Hispanic, non-Hispanic White, non-Hispanic Black, and non-Hispanic Asian), and the ratio of family income to poverty (poverty)^74^. This ratio was calculated by dividing the family income by the poverty guidelines for the survey year^74^. Furthermore, BMI and waist circumference were measured. The lipid test indicators included high-density lipoprotein cholesterol (HDL-C) and total cholesterol. The questionnaire data included receiving blood, the hepatitis B vaccine, general health condition, and using needles to inject illegal drugs (illegal drug injection). The 12 predictors that were used in our study were selected based on HBV and HCV guidelines.

For preprocessing and normalization, we followed the following procedures as detailed. NHANES data collection employs a multistage probability sampling design, ensuring a robust and representative dataset for disease prediction and healthcare planning^31^. “Refused” and “Don’t know” values were categorized as missing values to prevent potentially misleading predictions. All cases with missing data for hepatitis and variables with 50% or more missing data were excluded to prepare the data. Outliers were detected using the IQR method and then capped within the 25th to 75th percentile range^75^. We perform preprocessing by grouping variables with similar semantic meaning into the same category. For instance, preprocessing involves encoding categorical features into a set of binary values to indicate the presence or absence of some category. For the NHANES dataset, which comprise a mixture of numerical and non-numerical features, preprocessing tasks are typically transform non-numerical variables into a format suitable for analysis. The variables in the NHANES dataset are one-hot encoded into multiple binary categorical variables where each variable (e.g., “Mexican American”, “Other Hispanic”) for the different race has a binary value of 0 and 1. These binary values serve to identify whether each subject falls into their respective category for each variable. One-hot encoding was performed on categorical data to implement a machine learning model based on a dataset with a total of 17 characteristics.

### Evaluation of diabetes and hepatitis

Diabetes was defined as having a fasting plasma glucose (FPG) ≥ 126 mg/dL, hemoglobin A1c (HbA1c) ≥ 6.5%, or a self-reported diabetes diagnosis during the interview^3^.

The participants were divided into two groups: HBV or HCV and non-HBV or non-HCV. The participants were labeled as having HBV or HCV if they answered “Yes” to hepatitis B or C infection, as represented by the question “Has a doctor or other health professional ever told you that you have hepatitis B?” or “Has a doctor or other health professional ever told you that you have hepatitis C?” If the participant answered “No” to all HBV and HCV conditions, the participant was labeled as not having HBV or HCV infection.

### Statistical analysis

The continuous variables were represented as the means with standard deviations (SD, normal distribution), and the categorical variables were represented as percent-ages with frequencies. We used the SMOTE to counteract the imbalance in the number of hepatitis and non-hepatitis subjects^76^.

SMOTE is an oversampling algorithm that generates additional samples based on the original dataset by setting a specific scale to balance the dataset using over-sampling methods^76^. The importance of the predictors was evaluated and plotted using an importance score to determine the best-performing model. All analyses were performed using the R statistical software 4.12 (The R Foundation for Statistical Computing, USA).

To detect outliers, the study utilized the interquartile range (IQR) method. Outliers were defined as values that exceeded the thresholds of 75th percentile + 1.5 × IQR or were lower than the 25th percentile—1.5 × IQR^75^. Once the outliers were identified, they were capped within the range of the 25th and 75th percentiles.

### Machine learning model evaluation

We evaluated four machine-learning models to predict the risk of HBV or HCV infection among patients with diabetes: RF, SVM, XGBoost, and LASSO, which were shown higher performance metrics compared to other algorithms in previous studies for predicting outcomes related to hepatitis^26,29,34,69^. To achieve the best performance, we conducted hyperparameter tuning for each algorithm (Table 4). This included exploring different values for the hyperparameters of the dataset and selecting the ones that provided the most favorable outcomes. We employed a randomized search with ten iterations of three-fold cross-validation to identify the optimal hyperparameters for each of the four models. We tuned the hyperparameters of each algorithm to achieve the best performance. Cross-validation, in which 70% of the data were used to train the model and 30% were used in the prediction test, was performed to validate the prediction effects. The confusion matrix and AUC-ROC were used to select the best prediction model.

**Table 4:** Search range for hyperparameter tuning.

Algorithm	Hyperparameters	Range of value	Optimal values
RF	mtry	1 to 10	4
	ntree	100 to 1000	324
SVM	kernel	[‘linear’, ‘polynomial’, ‘radial’, ‘sigmoid’]	‘linear’
	cost	0.1 to 10	7.790
	degree	[3–5] except ‘linear’	
XGBoost	eta	0.01 to 0.1	0.089
	gamma	0 to 1	0.129
	max_depth	1 to 10	8
	nrounds	10 to 100	40
LASSO	alpha	0 to 1	0.013
	lambda	0 to 1	0.008

RF, random forest; SVM, support vector machine; XGBoost, extreme gradient boosting; LASSO, least absolute shrinkage and selection operator.

The performance metrics included the accuracy, sensitivity, specificity, precision, area under the curve (AUC), and F1 score. As the AUC tended to screen for degradation, we considered the sensitivity and specificity in conjunction with the AUC to minimize unbalanced bias^77^. The sensitivity is the ratio of true negativity that is accurately identified by the test and the specificity is the ratio of true negativity that is correctly identified by the test^78^. The F1 score is a harmonic mean of the accuracy and precision, which enabled us to compare different model performances in identifying actual disease predictions compared to false positives^79^. The importance of the predictors was evaluated and computed using the contribution importance rank of each variable to determine the best-performing model.

## Data Availability

All data in the current analysis are publicly available on the NHANES website (http://www.cdc.gov/nchs/nhanes.htm).
